# Database and geospatial mapping study of those eligible for extracorporeal cardiopulmonary resuscitation in the Thames Valley Region, England

**DOI:** 10.1016/j.resplu.2025.100879

**Published:** 2025-01-27

**Authors:** Oscar Millerchip, Jasper Eddison, Alex Rosenberg, Jon Bailey, James Raitt

**Affiliations:** a National Heart and Lung Institute, Faculty of Medicine, Imperial College London, Guy Scadding Building, Cale Street, London SW3 6LY, United Kingdom; b Harefield Hospital, Guys’ and St Thomas’ NHS Foundation Trust, Hill End Road, Harefield, Uxbridge UB9 6JH, United Kingdom; c Thames Valley Air Ambulance, Stokenchurch House, Oxford Road, Stokenchurch, High Wycombe HP14 3SX, United Kingdom

**Keywords:** Pre-hospital, Refractory cardiac arrest, Extracorporeal cardiopulmonary resuscitation

## Abstract

**Background:**

Out-of-hospital cardiac arrest survival remains low. Extracorporeal-cardiopulmonary resuscitation (ECPR) is a therapy for refractory out-of-hospital cardiac arrest that can improve survivability by decreasing the time a patient is without adequate perfusion, the low-flow time. Access to ECPR is limited by the number, location and delivery approach of centres offering this therapy.

**Aims:**

This study aims to identify how many patients are eligible for ECPR in the Thames Valley area and provide geographical analysis to appraise the specialist-centre approach of ECPR delivery in the region.

**Methods:**

Data from out-of-hospital cardiac arrests attended by the Thames Valley Air Ambulance from 1st Jan 2022 to 1st Jan 2024 were reviewed for eligibility to receive ECPR against inclusion criteria. Eligible cases were modelled using Geographic Information System software, and spatial autocorrelation analysis was performed to identify any significant ‘hotspots’, ‘cold spots’, or significant geographical distribution of eligible cases.

**Results:**

Of some 1,182 cardiac arrests attended, 188 (16%) cases were eligible under inclusion criteria for ECPR. In 2023 seven patients received ECPR, all focussed in a small area of the Thames Valley. The majority of eligible cases fall outside of the catchment of any one hospital when utilising the hospital-based or rendez-vous models of ECPR. Global Moran’s analysis of the entire region found no significant clustering or dispersal, suggesting a near-random distribution despite some evidence of hotspots.

**Conclusion:**

ECPR can improve survival for out-of-hospital cardiac arrest, but time constraints preclude access to this therapy for many, which affects equitability across a geographical area. Geospatial analysis techniques can aid in reviewing the optimal delivery methods of ECPR and improve equitable geographical access to services. The methodology described may aid other organisations in planning the delivery of ECPR.

## Background

### ECMO and ECPR

Out-of-hospital cardiac arrest (OHCA) has a poor prognosis and is an ongoing challenge for pre-hospital emergency care providers. Despite advances in pre-hospital care and broader education on cardiopulmonary resuscitation (CPR), mortality rates remain high at 91–93%.^1^ CPR efficacy quickly declines and the longer a patient is in cardiac arrest, the less likely they are to survive. Beyond 30 min of conventional CPR survival rates to hospital discharge approach 0%.^2^ Cardiac arrests are refractory when a return-of-spontaneous-circulation (ROSC) is not achieved despite resuscitation attempts.^3^

Extracorporeal membrane oxygenation (ECMO) is a system that provides circulatory support by draining blood, oxygenating it, and returning it to the patient. Veno-arterial ECMO is used for cardio-respiratory support where blood is drained from the venous system and returned to the arterial system, thereby maintaining end-organ perfusion.^4^

Extracorporeal cardiopulmonary resuscitation (ECPR) is the utilisation of VA ECMO for patients in refractory cardiac arrest, performed alongside high-quality advanced life support (ALS).^3^ Survival to hospital discharge for OHCA patients with any attempted resuscitation is 7–9% in the South-Central region of the United Kingdom.^1^, ^5^, ^6^ In patients who receive ECPR, survival to hospital discharge can likely increase to 23–25%^3^, ^7^ and by >30% as reported in the Extracorporeal Life Support Organisation’s world registry for adult ECPR outcomes.^8^

ECPR is considered a high-acuity, low-occurrence procedure, where a clear volume-outcome relationship is established for ECMO.^9^ ECPR is a complex intervention, requiring high-performing teams,^10^, ^11^ patient selection^12^ and the minimisation of low-flow time^13^, ^14^ to improve survival rates.

Minimising low-flow times presents significant logistical challenges to providing ECPR. Patients benefit most when ECPR is deployed within 60 min of cardiac arrest.^15^, ^16^, ^17^, ^18^ Various models of ECPR are used, including transporting a patient to a specialist-centre for ECMO,^11^, ^19^, ^20^ deploying ECMO pre-hospital in the field,^21^ or an ECPR team rendezvous at a non-specialist hospital to initiate ECMO.^22^, ^23^

The Harefield Hospital’s ECPR service was redeveloped in 2023 when they entered a partnership with TVAA. Under the current model patients are extricated to the Harefield Hospital if their transport time is less than 30 min and their arrival time to the hospital is within one hour of collapse, however this raised the question of how equitable ECPR is geographically. Methodologies are previously described to assess ECPR services and highlight the value geographical modelling to inform service provision.^22^, ^24^, ^25^, ^26^, ^27^, ^28^, ^29^ Further, this study utilises both local and global (whole area) spatial autocorrelation methods^30^ described in other fields^31^, ^32^ to explore overall geographical distributions, regional ‘hotspots’ and give insight to the efficacy of different ECPR deployment methods. The methodology described may be used by other services wishing to build a business case and implement their own ECPR service and assess the optimal approach to delivering ECPR to their own patient cohort.

#### Aims

This study aims to identify how many patients are eligible for ECPR and uses geographical analysis to appraise this specialist-centre approach of ECPR deployment for all patients in the Thames Valley.

## Methods

### ECPR service

#### Pre-hospital

TVAA operate an Airbus EC135 helicopter alongside critical care response vehicles. The entirety of the Thames Valley can be reached within 15 min by air, discounting required pre-flight preparation time. Cardiac arrest patients are often extricated by road owing to restricted patient access in the helicopter.^19^ The helicopter or response vehicles are used to transport clinicians and equipment to the scene.

TVAA work alongside the South-Central Ambulance Service (SCAS) covering the Thames Valley. A critical care paramedic and an emergency dispatch assistant interrogate and triage 999 calls for TVAA assistance.^19^ Calls identified as potential ECPR candidates are identified and patients are assessed on-scene with on-going high-quality ALS. If conveyance of the patient to the Harefield Hospital is possible within 60 min of their OHCA, they are immediately extricated to the Harefield Hospital by a SCAS ambulance with ongoing CPR.

#### In hospital

**Fig. 1:**
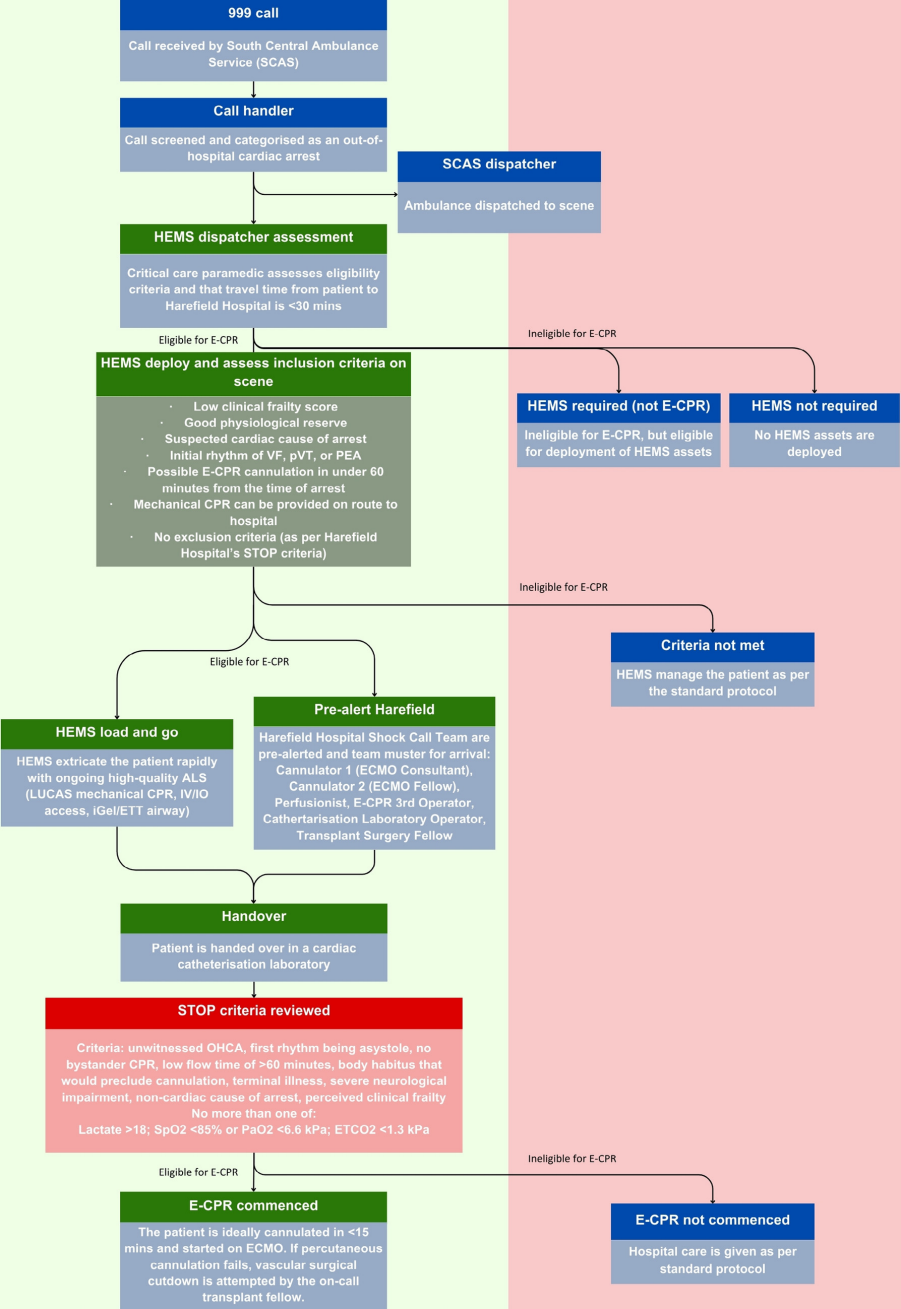
A summary of deciding ECPR eligibility from 999 call to initiation of ECMO. VF, ventricular fibrillation; pVT, pulseless ventricular tachycardia; PEA, pulseless electrical activity; ALS, advanced life support; IV/IO, intravenous/intraosseous; ETT, endotracheal tube).

The Harefield Hospital is a quaternary, specialist cardiac centre with heart and lung transplant capabilities and a commissioned ECMO service. Patients are handed over in a dedicated cardiac catheterisation laboratory, where in-hospital STOP criteria for initiation of ECPR are re-evaluated, as in Fig. 1. If eligible, the ECPR team will aim to cannulate the patient and commence ECMO within 15 min of arrival.

### Study setting

TVAA serve the rural and semi-rural counties of Oxfordshire, Buckinghamshire and Berkshire (calls out-of-area are excluded). The population of the Thames Valley was approximately 2,550,000 in mid-2022.^33^ The Harefield Hospital is located outside the Thames Valley, to the Southeast of the region.

The region was mapped and divided into the three counties, using ceremonial county lines as per Ordnance Survey,^34^ and subdivided using outcodes. Outcodes are the initial characters of a postcode before the break, for example the outcode for SW1A 1AA is SW1A, and allow analysis of smaller localities without making OHCAs identifiable. When county lines transect outcodes, the entire outcode is included for numerical analysis.

Hospitals in and around the Thames Valley offering 24 h a day percutaneous coronary intervention (PCI) are the Harefield, Wexham Park, Royal Berkshire, and John Radcliffe,^35^ which are included in this study. Centres offering PCI in-hours, Milton Keynes, the Great Western, and Wycombe Hospitals are included. The only hospital offering ECMO is the Harefield Hospital.

### Study population

This study includes all OHCA in the Thames Valley region attended by TVAA. Eligibility of OHCA is assessed from 1 January 2022 to 1 January 2024. TVAA’s service operates from 0700 to 0200, 365 days a year. The Harefield Hospital and TVAA’s ECPR service partnership began on 1 January 2023. Cases with initiated ECPR are from 1 January 2023 to 1 January 2024.

**Fig. 2:**
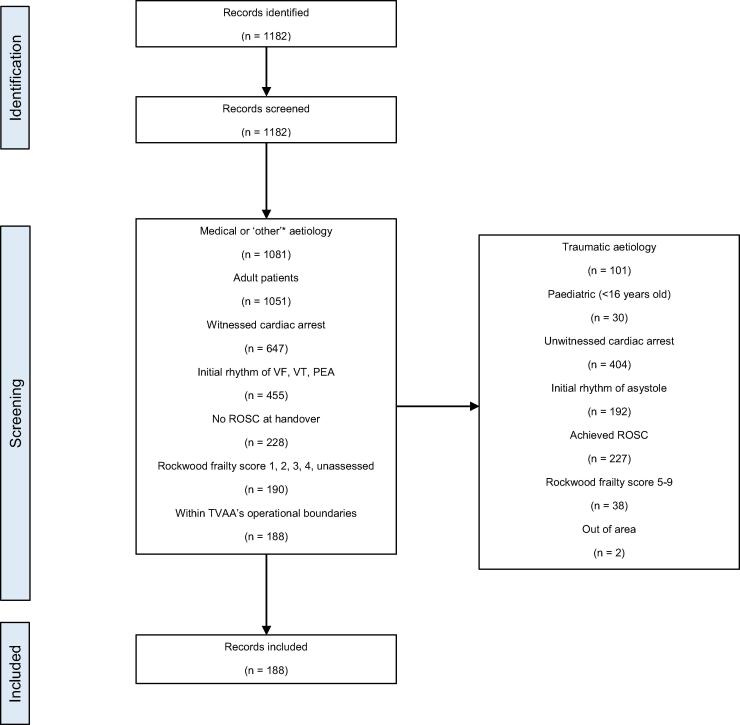
A PRISMA diagram showing eligibility screening of cases. Records were identified, screened as per inclusion criteria, and either included or excluded.

Patient data were extracted from HEMSBase, anonymised, and filtered using the TVAA inclusion criteria for ECPR, shown in Fig. 2.^36^ Included cases were adult (>16 years of age) cardiac arrests of a medical aetiology, that were witnessed at the time of arrest and received bystander CPR. Included cases were assessed by TVAA on arrival to be in an initial rhythm of either ventricular fibrillation (VF), pulseless ventricular tachycardia (pVT), or pulseless electrical activity (PEA), without sustained ROSC, and with a Rockwood clinical frailty score (RCFS) of 1, 2, 3, 4, or unassessed. ROSC at handover was used as a surrogate marker for sustained ROSC. The RCFS is used to assess the perceived frailty and physiological reserve in a manner more useful than an absolute age cutoff. Patients younger than 60 do not have their RCFS assessed but are included. Under the TVAA/Harefield Hospital’s current specialist-centre approach, only cases that can reach the Harefield Hospital within 60 min of OHCA are considered ‘eligible’. OHCAs outside of a 30-minute drive time from the Harefield Hospital are included to review differing methods of ECPR delivery and assess the patient cohort of potentially eligible patients across the entire region, regardless of ECPR delivery method. Patients with missing or incomplete data were searched by a TVAA pre-hospital emergency medicine consultant and categorised.

Characteristics of all OHCAs, and subgroups of eligible and ineligible for ECPR were collated and summarised in Table 1.

**Table 1:** All out-of-hospital cardiac arrests, ineligible for ECPR, and eligible for ECPR cases categorised by age, sex, aetiology of cardiac arrest, if the cardiac arrest were witnessed, the initial rhythm, and the patient’s frailty score as measured with the Rockwood Clinical Frailty Scale.

Characteristics	All out-of-hospital cardiac arrests	Ineligible for ECPR	Eligible for ECPR
Number of patients (percentage of all OHCAs, %)	*n* = 1182	*n* = 994 (84)	*n* = 188 (16)
Age, median (Q_1_–Q_3_)	62 (50–72)	62 (48–72)	64 (55–73)
Sex (%)			
Male	842 (71)	699 (70)	143 (76)
Female	336 (28)	291 (29)	45 (24)
Other	4 (<1)	4 (<1)	0 (0)
Aetiology (%)			
Medical	1054 (89)	866 (87)	188 (100)
Trauma	99 (8)	99 (10)	0 (0)
Other	25 (2)	25 (3)	0 (0)
Unknown	4 (<1)	4 (<1)	0 (0)
Witnessed (%)			
Yes	711 (60)	523 (53)	188 (100)
No	471 (40)	471 (47)	0 (0)
Initial rhythm (%)			
Shockable			
VF	325 (27)	230 (23)	95 (51)
VT	21 (2)	16 (2)	5 (3)
Unknown	7 (<1)	4 (<1)	3 (2)
Non-shockable			
PEA	249 (21)	168 (17)	81 (43)
Asystole	531 (45)	531(53)	0 (0)
Unknown	49 (4)	45 (5)	4 (2)
Frailty score (%)			
1–2	206 (17)	172 (17)	34 (18)
3–4	289 (24)	214 (22)	75 (40)
5–9	142 (12)	142 (14)	0 (0)
Unassessed	545 (46)	466 (47)	79 (42)

### Mapping

Geographical data from EDINA’s Digimap, Edinburgh, UK, using Ordinance Survey data from July 2023, were sourced and compiled.^34^ These were mapped using ESRI’s ArcGIS Pro, Version 10.8.2 for Windows 10, California, USA. Postcodes were truncated to outcodes using Python, Version 2.7.18.4 for Windows 10, Virginia, USA. Population data were sourced from the Office for National Statistics, using National Census data from 2021,^37^ compiled into outcodes using Excel, Version 16.77.1 for macOS, Washington, USA. Incidence calculations used mid-2022 population estimates by Lieutenancy (ceremonial) areas from the Office for National Statistics.^33^

*Incidence of overall OHCA and ECPR eligible OHCA.* The mean annual incidences were calculated using both years’ data, per 100,000 population.

*Quantile analysis.* Data were split into eight classes of equal frequency and represented in a gradient.

### Geospatial analysis

To identify geographical equitability, data were analysed using geospatial autocorrelation techniques. This way, local areas of significantly high and low-volume are identified and the overall (global) region is summarised in terms of eligible case distribution. These results combined with drive-time isochrones allow inference to the equitability and expected patient cohort of different deployment methods of ECPR for local ‘hotspots’ and for the region overall. The results for local analyses using local Moran’s I and Getis-Ord Gi* are combined to increase reliability. For these analyses, distances used throughout are Euclidean (straight-line) rather than road based. These analyses use 499 permutations when processing significance levels (accounting for expected clustering due to chance) with significance values precise to 0.002. Methods of analyses are described below, with greater detail in Appendix A.

*Anselin local Moran’s I*. Measures similarity across an area, identifies significant clusters, and categorises them by type of ‘hot’ or ‘cold’ spot.^38^ Moran’s analyses were performed for the frequency of cases and the frequency of cases per 100,000 population and mapped.

Getis-Ord Gi*. Measures distribution over area in ‘hotspots’ and ‘coldspots’.

*Global Moran’s I.* Quantifies significant clustering or dispersal as a summary statistic of the entire region. Perfectly clustered data have a Moran’s index of +1.0, perfectly dispersed data have a Moran’s index of −1.0, and completely random data have a Moran’s index of 0.^38^

*Isochrone mapping.* Isochrones are a 30-minute drive time analysis of non-blue light driving in average traffic conditions, performed in ArcGIS Online/ArcGIS Pro. The area in km^2^ of each hospital’s drive time isochrone (DTI) was calculated using ArcGIS Pro, and the population encompassed inside each DTI was approximated. The population within each outcode was included in the total if >50% of the outcode fell geographically within the DTI.

### Ethics

The NHS Health Research Authority’s decision tool deemed formal ethical approval for this project unnecessary.^39^ Patient data were extracted from HEMSBase by TVAA’s data guardian and anonymised. Anonymised patient data were stored securely on an encrypted, password-protected cloud.

## Results

Of 1,182 out-of-hospital cardiac arrests in the Thames Valley region attended by TVAA, 188 (16%) were potentially eligible for ECPR, meeting pre-hospital inclusion criteria outlined in Fig. 1. The mean annual incidence of OHCA attended by TVAA was 23.2 per 100,000 population. The mean annual incidence of ECPR eligible OHCA was 3.7 per 100,000 population. Characteristics of OHCAs attended by TVAA are summarised in Table 1. In 2023, seven patients received ECPR.

### Eligible versus ineligible for ECPR

The median age for ECPR eligible patients was 64, and 62 for ECPR ineligible patients. Eligible cases had a lower interquartile range of 18 years, compared to 24 for ineligible cases. OHCAs were more common in males overall (71% male), in the ECPR ineligible group (70% male) and the ECPR eligible group (76% male).

### Characteristics from inclusion criteria

Of all cardiac arrests, 89% were of medical aetiology, 97% were > 16 years of age, 60% were witnessed, 50% presented in an initial rhythm of either VF, VT, or PEA, 71% did not sustain ROSC at handover, and 87% presented with a RCFS of 1, 2, 3, 4, or unassessed.

### Geospatial results

**Fig. 3:**
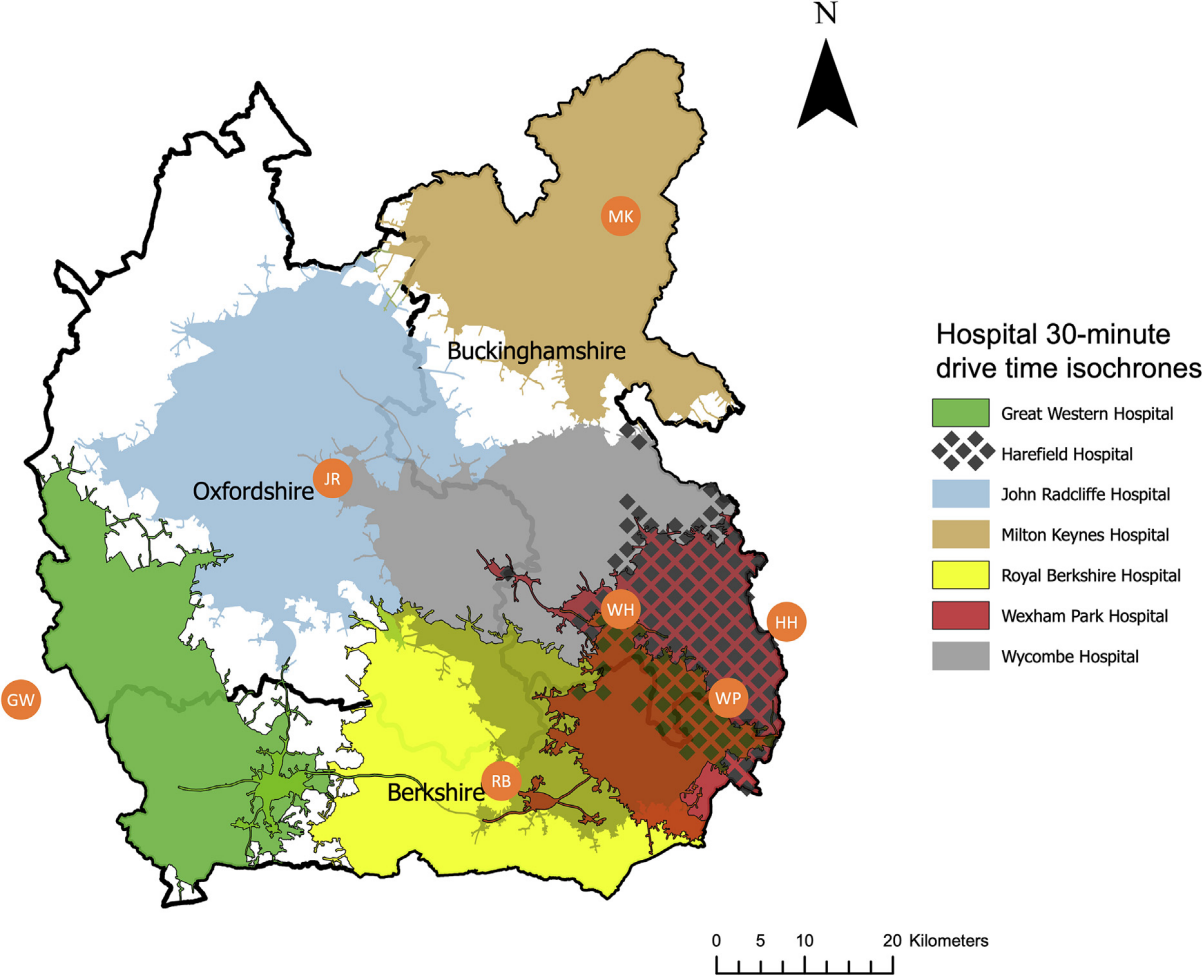
Hospital 30-minute drive time isochrones in the Thames Valley. Hospitals are marked with orange circles: Harefield, HH; Wexham Park, WP; Wycombe, WH; Royal Berkshire, RB; Milton Keynes, MK; John Radcliffe, JR; Great Western, GW. A thick black line outlines the county borders, labelled. A scale bar (km) is shown. (For interpretation of the references to colour in this figure legend, the reader is referred to the web version of this article.)

Fig. 3 outlines the geographical area of the Thames Valley and the major hospitals offering PCI.^35^ Non-blue light 30-minute drive time isochrones (DTI) to each hospital are overlayed based on average traffic and road conditions.

The total area of the Thames Valley is 5743 km^2^ with ceremonial county borders. In all, 4543 km^2^ (79%) of the Thames Valley is within a DTI of at least one hospital, accounting for approximately 2,226,600 people (87% of the Thames Valley population). The total area outside any DTI is 1200 km^2^, accounting for approximately 323,000 people (13% of the Thames Valley population). Of the ECPR eligible OHCAs, 156 (83%) occurred within a hospital DTI.

The total geographical areas covered by each hospital’s DTI are: Wycombe (WH), 1492 km^2^; John Radcliffe (JR), 1325 km^2^; Royal Berkshire (RB), 1136 km^2^; Milton Keynes (MK), 816 km^2^; Great Western (GW), 689 km^2^; Wexham Park (WP), 651 km^2^; Harefield (HH), 478 km^2^.

The estimated populations within each hospital’s DTI are: Wycombe, 878,000; Royal Berkshire, 876,000; Wexham Park, 579,000; Harefield, 413,000; Milton Keynes, 405,000; John Radcliffe, 388,000; Great Western, 143,000.

The estimated number of ECPR eligible OHCA inside each hospital’s DTI is 77 (41%) for Wycombe Hospital, 72 (38%) for the Royal Berkshire, 51 (27%) for Wexham Park, 32 (17%) for the Harefield, 27 (14%) for the John Radcliffe, 22 (12%) for Milton Keynes and 5 (3%) for the Great Western Hospital.

**Fig. 4:**
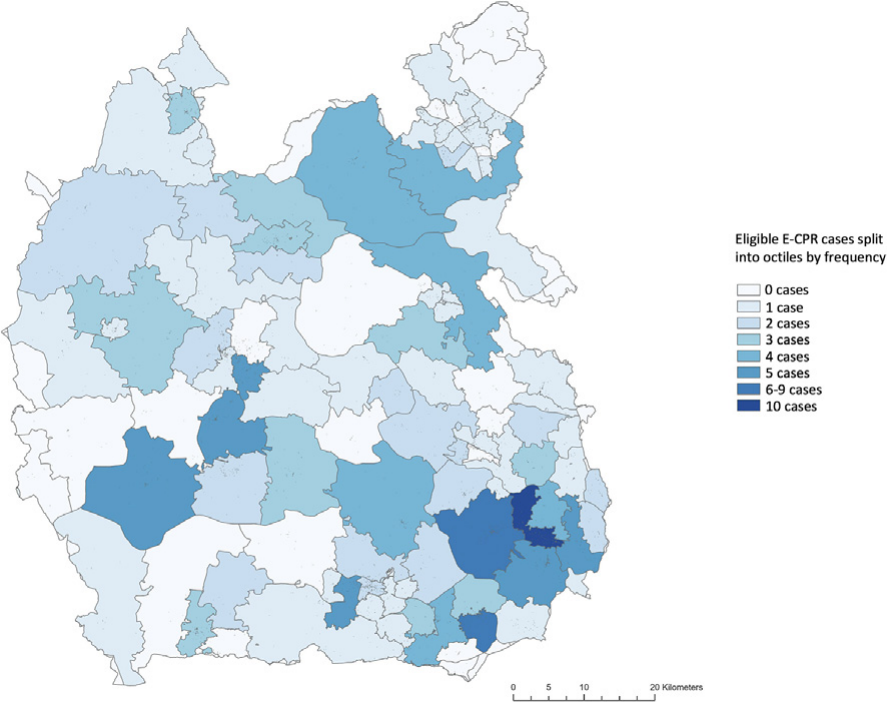
Eligible ECPR cases split into eight classes. Darker blue represents more cases. A scale bar (km) is shown. (For interpretation of the references to colour in this figure legend, the reader is referred to the web version of this article.)

Fig. 4 shows eligible cases for ECPR across the region.

**Fig. 5:**
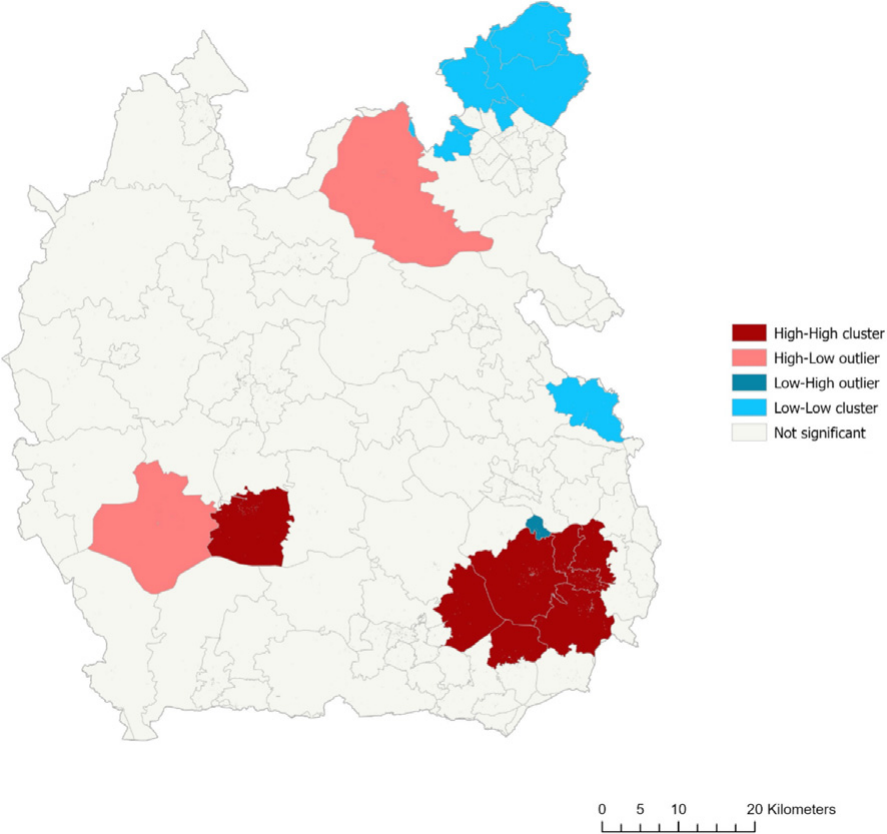
Anselin Moran’s I local analysis of ECPR eligible cases. Outcodes are displayed in light grey. Dark red signifies high-high clusters, light red signifies high-low outliers, light blue signifies low-low clusters and dark blue signifies low–high outliers. Off-white signifies areas of no significance. A scale bar (km) is shown. (For interpretation of the references to colour in this figure legend, the reader is referred to the web version of this article.)

Fig. 5 displays the output of Moran’s local I analysis of ECPR eligible cases (unadjusted for population) that were significant to the *p < 0.05* significance level. High-high, high-low, low–high and low-low clusters were identified. Getis-Ord Gi* analysis equally identified hotspots and cold spots unadjusted for population, but only hotspots when controlled for population.

The outcodes SL1 and SL6 were identified as significant hotspots by both analysis methods. When adjusted for population, no significant hotspots or cold spots were identified in the Thames Valley.

Global Moran’s I index for the Thames Valley is 0.0729 (*p > 0.05*), indicating a non-significant, closer-to-random distribution of ECPR eligible cases.

The seven eligible cases that received ECPR were all inside the Harefield Hospital’s DTI.

## Discussion

### Eligibility

In the Thames Valley, 16% of OHCA are eligible for ECPR under inclusion criteria in 2022 and 2023. Whilst the minority of cases are eligible, this proportion represents a significant absolute number of eligible patients. Of these eligible cases, seven patients were within the Harefield Hospital’s DTI and extricated to the Harefield Hospital to receive ECPR under the TVAA/Harefield Hospital partnership in 2023. All cases were within the Southeast of the region.

Eligibility rates are intrinsically linked to how liberal selection criteria are. Rates appear comparable to similar eligibility studies in the UK and globally and are informed by current data.^25^, ^40^, ^41^, ^42^

### Geographical equitable access

When combined, Moran’s local I and Getis-Ord Gi* analyses identified two significant hotspots. Both hotspots lie geographically within the Harefield Hospital’s DTI. When controlled for population, no significant hotspots or cold spots were identified. This is supported by Global Moran’s I statistic of 0.0729 indicating a close-to-random distribution of eligible cases across the region. As such, attention should be given to the entire region, rather than concentrating resources around high-volume areas. In the case of significant hot spots and cold spots, it is expected that Global Moran’s may be closer to +1.0 where concentrating resources may be more beneficial.

Of 188 potentially ECPR eligible cases, 32 occurred inside the Harefield Hospital’s DTI. Most (83%) eligible cases occurred within at least one hospital’s DTI, with 32 (17%) cases occurring outside of all hospital’s DTI. The hospital with the largest geographical area, population served within the Thames Valley, and proportion of eligible cases was Wycombe Hospital.

The Harefield Hospital and Wycombe Hospital are ideally located to serve these ‘hotspots’, although only the Harefield Hospital offers ECMO in the area. Considering a near-random distribution of eligible cases across the region, a different method of delivering ECPR would likely improve the number of cases receiving ECPR. The largest barrier to equitable ECPR access in the Thames Valley is geographical location, given both the majority of eligible cases and the majority of the population reside outside of the Harefield Hospital’s DTI.

### Approach to ECPR delivery

The method of deploying ECPR to a population should ideally be tailored to the infrastructure, geography, and case-volume of a region. For the TVAA/Harefield Hospital’s specialist-centre approach, ECMO operators are highly experienced which contributes to increased survival rates.^9^ However, it means a small subset of eligible patients receive this higher level of care. Spatial autocorrelation analysis and geographical modelling suggest that a specialist-centre approach for this population is geographically inequitable.

Alternative deployment methods may be better suited to this population. Given a more uniform distribution across the Thames Valley, a ‘rendezvous’ approach of extricating a patient to a non-specialist hospital, commencing ECPR there, and repatriating patients to a specialist-centre would cover an estimated 83% of the Thames Valley by relying on an existing network of major hospitals. Though evidence is limited, this approach is described elsewhere^23^ and circumvents the high-acuity, low-volume of patient issues when setting up new specialist-centres and trying to achieve international standards in ECPR care.^43^

ECPR can be deployed in the field.^44^, ^45^ Evidence for this approach is limited and no increase in survival rates to hospital discharge are described, but low-flow times were decreased.^46^ A helicopter-deployed service is also described in Paris, where an ECMO team are loaded and deployed by air to a patient, giving access to ECPR for patients who might otherwise have little access to any meaningful medical intervention.^21^ The entirety of the Thames Valley can be reached within 15 min by air, and a helicopter-deployed ECPR service would make the whole region geographically accessible within the 60-minutes of low-flow time window. These approaches are supported by predictive modelling,^47^ however, more data are required to validate these deployment methods.

### Limitations

This paper demonstrates geospatial analysis as a useful tool when considering equitable access to services. Isochrone mapping is also useful but may overestimate drive times, given the dynamic nature of traffic. These analyses are limited by the dataset. ECPR cases are skewed towards the Harefield Hospital and areas near deployable cars because of the time-sensitive nature of ECPR. Ambulance service data could provide a more complete model of all OHCA. The subdivision of areas being as large as outcodes reduces the reliability of geospatial analysis. For Moran’s analysis to function optimally, each area should have eight or more neighbours, especially in skewed data, so Moran’s Global I and Local I are likely overexpressed. Smaller land divisions may be appropriate where patient confidentiality allows. This paper focuses on the pre-hospital screening of OHCAs. In-hospital exclusion criteria, though very similar, were not used to select ‘eligible’ candidates. Further cases may be excluded based on physiological STOP criteria requirements.

## Conclusion

In the Thames Valley, 16% (188) OHCAs were eligible for ECPR. In 2023, seven cases were successfully initiated on ECPR. Currently, the TVAA/Harefield Hospital’s ECPR service covers a small geographical area and leaves most of the Thames Valley underserved. Using one method of geospatial analysis revealed significant hotspots, but combining methods found no significant hot spots and a uniform distribution of eligible cases across the region. A multimodal approach to geospatial analysis increases reliability and is more informative to the likely benefit of each ECPR deployment method. The challenge of early initiation in deploying ECPR is highlighted here and emphasises the need for a wide reaching and rapid means of deployment, to ensure equitable geographical access to ECPR.

## CRediT authorship contribution statement

**Oscar Millerchip:** Writing – original draft, Visualization, Software, Methodology, Formal analysis, Conceptualization. **Jasper Eddison:** Writing – original draft, Visualization, Methodology. **Alex Rosenberg:** Writing – review & editing, Supervision, Methodology, Conceptualization. **Jon Bailey:** Writing – review & editing. **James Raitt:** Writing – review & editing, Supervision, Methodology, Data curation, Conceptualization.

## Funding

Funding is provided by the Imperial Open Access fund. The funder had no role in the conceptualisation, design, data analysis, preparation or decision to publish this manuscript.

## Declaration of competing interest

The authors declare that they have no known competing financial interests or personal relationships that could have appeared to influence the work reported in this paper.

## Appendix A

*Anselin local Moran’s I*. Moran’s test, or Local Indicators of Spatial Association, measures similarity across an area. In this, clusters are formed and displayed if they reach statistical significance*.* They are summarised in four groups, depending on how the areas surround them are categorised. High-high clusters are areas of increased frequency surrounded by other areas of increased frequency, whereas high-low clusters are areas of increased frequency surrounded by areas of respectively lower frequencies. The converse is true for low-low clusters and low–high outliers, ‘coldspots’.^38^

*Global Moran’s I.* A global Moran’s test, a form of spatial autocorrelation, is a summary statistic of the frequency spread relative to geolocation and summarises if there is clustering or dispersion in the data. Perfectly clustered data have a Moran’s index of +1.0, perfectly dispersed data have a Moran’s index of −1.0, and ‘perfectly random’ data have a Moran’s index of 0.^38^

*Getis-Ord Gi*.* Getis-Ord Gi* is another spatial analysis that measures distribution over area. To identify a ‘hotspot’, an area must be surrounded by other higher-frequency areas. The inverse is true for ‘coldspots’. The identified ‘hotspots’ and ‘coldspots’ are then stratified according to their significance level.
